# Seeing through sedimented waters: environmental DNA reduces the phantom diversity of sharks and rays in turbid marine habitats

**DOI:** 10.1186/s12862-021-01895-6

**Published:** 2021-09-06

**Authors:** Yin Cheong Aden Ip, Jia Jin Marc Chang, Kelvin K. P. Lim, Zeehan Jaafar, Benjamin J. Wainwright, Danwei Huang

**Affiliations:** 1 Department of Biological Sciences, National University of Singapore, 16 Science Drive 4, Singapore, 117558 Singapore; 2 Lee Kong Chian Natural History Museum, National University of Singapore, 2 Conservatory Drive, Singapore, 117377 Singapore; 3 Yale-NUS College, National University of Singapore, 16 College Avenue West, Singapore, 138527 Singapore; 4 Centre for Nature-based Climate Solutions, National University of Singapore, 16 Science Drive 4, Singapore, 117558 Singapore; 5 Tropical Marine Science Institute, National University of Singapore, 18 Kent Ridge Road, Singapore, 119227 Singapore

**Keywords:** 12S ribosomal RNA, Chondrichthyes, Cytochrome *c* oxidase subunit I, Dark diversity, Southeast Asia, Urban coastlines

## Abstract

**Background:**

Sharks and rays are some of the most threatened marine taxa due to the high levels of bycatch and significant demand for meat and fin-related products in many Asian communities. At least 25% of shark and ray species are considered to be threatened with extinction. In particular, the density of reef sharks in the Pacific has declined to 3–10% of pre-human levels. Elasmobranchs are thought to be sparse in highly urbanised and turbid environments. Low visibility coupled with the highly elusive behaviour of sharks and rays pose a challenge to diversity estimation and biomonitoring efforts as sightings are limited to chance encounters or from carcasses ensnared in nets. Here we utilised an eDNA metabarcoding approach to enhance the precision of elasmobranch diversity estimates in urbanised marine environments.

**Results:**

We applied eDNA metabarcoding on seawater samples to detect elasmobranch species in the hyper-urbanised waters off Singapore. Two genes—vertebrate 12S and elasmobranch COI—were targeted and amplicons subjected to Illumina high-throughput sequencing. With a total of 84 water samples collected from nine localities, we found 47 shark and ray molecular operational taxonomic units, of which 16 had species-level identities. When data were compared against historical collections and contemporary sightings, eDNA detected 14 locally known species as well as two potential new records.

**Conclusions:**

Local elasmobranch richness uncovered by eDNA is greater than the seven species sighted over the last two decades, thereby reducing phantom diversity. Our findings demonstrate that eDNA metabarcoding is effective in detecting shark and ray species despite the challenges posed by the physical environment, granting a more consistent approach to monitor these highly elusive and threatened species.

**Supplementary Information:**

The online version contains supplementary material available at 10.1186/s12862-021-01895-6.

## Background

Sharks and rays are some of the most threatened marine taxa [1] due to their high demand as food fish [2], fin-related products in many Asian countries [3–5], Traditional Chinese Medicine [6], as well as high levels of bycatch [7]. Their slow growth (5–10 cm/year), late maturity (5–15 years), low fecundity (litter size < 100 per year), and high ecological risk [8, 9] exacerbate the threats posed by overfishing and further hinder population recovery [10]. At least 25% of assessed shark and ray species are considered to be threatened with extinction based on the IUCN Red List of Threatened Species [7]. Specifically, the present density of sharks has declined to 3–10% of pre-human levels within Pacific coral reefs [11], and they are functionally extinct in ~ 20% of reefs surveyed globally [12]. These megafauna therefore constitute a considerable portion of dark diversity—species that have been historically reported and still exist in the greater surrounds of their known geographic ranges, but are presently missing from a specific area [13]. They could also represent phantom diversity—extant species that are locally colonised but have become too rare to be detected by regular survey methods [14]. Failure to detect these large predators has conservation and management implications [15], and in some cases, learned avoidance behaviour of elasmobranchs further amplifies the challenges of monitoring these species [16].

The defaunation of sharks and rays have dire consequences for coral reef health [17, 18]. It can alter biological communities [19], with impacts cascading down trophic levels [20]. Apart from the loss of ecosystem services conferred by these organisms, the disappearance of these megafauna would impede our understanding of the drivers of species distribution and resilience, curtailing efforts for the formulation and implementation of management strategies [17].

Marine habitats are threatened by a wide range of anthropogenic-induced stressors such as climate change [21], overfishing [12], pollution [22], and habitat degradation [23, 24]. Collectively, these impacts have led to population declines and extinctions [14, 25]. These consequently either increase an area’s dark diversity due to extirpation of resident populations [13], or augment phantom diversity as these species may have left regular survey areas and are mistakenly regarded to be locally extinct [14]. From a conservation perspective, it is thus important to ascertain if non-detection is a result of the former or the latter because each has its own management implications. For instance, rediscovery of rare, phantom species can spur expansion of survey areas or improved techniques for monitoring success [14], while confirmed species losses might require further investigation into whether community- or ecosystem-level functions have been impacted and, if necessary, kickstart population recovery initiatives to allay further losses [13]. Clearly, the need to diversify survey methods for biomonitoring has never been greater.

Species detection can be a tall order in the marine realm, because these environments are comparatively less accessible and thus less well-studied compared to terrestrial environments [26]. Conventional biomonitoring methods, such as underwater visual census (UVCs) and baited remote underwater video stations (BRUVS), while fundamental for providing data to support the management of marine species, are time-consuming, labour- and cost-intensive [27]. These approaches may not always fare well for elusive fauna like low-density, highly-mobile sharks and rays [28]. Fortunately, recent advancements in DNA sequencing technologies [29, 30] have yielded new possibilities to work around present biomonitoring challenges. One such application is high-throughput sequencing (HTS) of organisms’ trace genetic material isolated from environmental samples, otherwise known as environmental DNA (eDNA) [31, 32]. This approach allows for the detection of species in the water without having to visually observe them [33], and has emerged as an effective non-invasive method for biomonitoring based on different sample types (e.g. sediment; [34]) from various aquatic environments ranging from freshwater [31, 35] to marine habitats [36, 37]. In particular, eDNA has become increasingly popular for monitoring sharks and rays [28, 38–41].

This is a welcomed development for areas with highly sedimented ecosystems as eDNA can help bypass the constant need for visual observations [42, 43]. One example is Singapore, where the highly urbanised and turbid waters have hindered in-water studies of marine fauna [43–45]. As such, in-situ shark and ray sightings are sparse and typically comprise chance encounters (Jaafar pers. obs.; [44]) or carcasses [45]. The poor water visibility (average Secchi depths of ~ 2 m; [46]) encumbers visual detection and limits the resolution of underwater surveys and video capture technologies [12, 28], thus increasing phantom diversity. Moreover, avoidance behaviour [16] in certain elasmobranchs further complicates biomonitoring efforts. As such, it remains unclear as to whether elasmobranch diversity in Singapore can be accurately estimated from historical records. A previous broad-based eDNA metabarcoding study only managed to recover a single elasmobranch species, *Carcharhinus melanopterus* [47]. Yet, many other shark and ray species have been reported in checklists (e.g. [48] for fishes in the eastern Johor Strait), as well as from angler reports and citizen science surveys [49]. There is undoubtedly a striking gap in our understanding of elasmobranch diversity in urbanised environments that can be filled by a more targeted eDNA approach.

To detect the diversity of elasmobranch species present in Singapore’s waters, this study targeted two genes—vertebrate 12S ribosomal RNA [50] and elasmobranch cytochrome *c* oxidase subunit I [28, 38]—from 84 water eDNA samples collected from nine localities for HTS. We compared our resulting eDNA data with historical collections and contemporary sightings to investigate the extent to which eDNA metabarcoding could uncover the phantom diversity of sharks and rays. Our findings not only help enhance our understanding of elasmobranch diversity in Singapore, they also demonstrate the utility of eDNA for studying mobile marine fauna in other turbid ecosystems. More broadly, this study and others will help inform biodiversity conservation and management practices by bringing eDNA methods closer to the routine biomonitoring of marine taxa and habitats [32, 51, 52].

## Results

### Illumina sequencing and primer efficiency

**Fig. 1:**
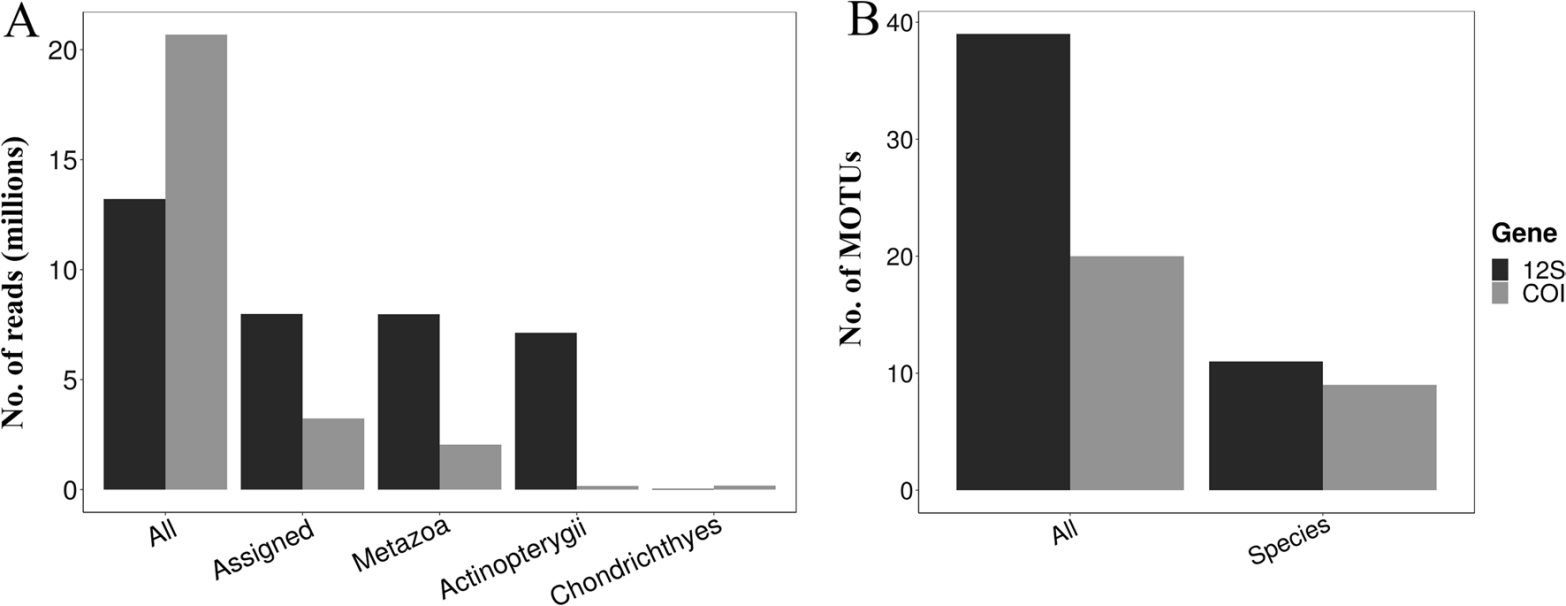
Unique sequence reads recorded from sequencing with vertebrate 12S (black) or elasmobranch COI (grey) primers. **A** Total number of unique sequence reads generated from each gene and the relative proportions identifiable to sequences from GenBank (‘Assigned’), from which they are segregated into the categories ‘Metazoa’, ‘Actinopterygii’ and ‘Chondrichthyes’. **B** Total number of Chondrichthyes MOTUs and named species units detected for each marker

Illumina sequencing collectively produced 515,657,226 paired-end assembled reads, of which we obtained 13,209,521 and 20,683,942 sequence reads (combined 33,893,463) from the 12S and COI sequencing respectively. Only 33.1% unique sequence reads (7,984,423 for 12S and 3,234,072 for COI) were identifiable against GenBank records. A total of 210,761 (30,777 for 12S and 179,984 for COI) unique sequence reads have closest matches (≥ 85%) to ‘Chondrichthyes’ (Fig. 1), making up 1.88% of the total unique reads sequenced. Similar statistics were obtained previously [28]. The rest of the unique sequence reads were mostly represented by ‘Actinopterygii’ and other ‘Metazoa’. Although 12S sequencing produced a higher proportion of Actinopterygii relative to unique Chondrichthyes reads, the vertebrate-specific primers yielded more Chondrichthyes species units than COI (Fig. 1).

We detected shark and ray species in all the water samples (*n* = 84) while none were found in the negative controls. The COI sequencing had generated more reads per sample and more consistent detection across PCR replicates than 12S sequencing (Additional file 1), especially for commonly detected species. Despite producing fewer total unique Chondrichthyes reads, the 12S primers detected more species than the COI primers, but at much shallower read depths across PCR replicates (Additional file 1). Detection of sharks with the 12S primers was observed to be less consistent among PCRs than the COI primer set.

### MOTU richness, phantom diversity and new records

**Fig. 2:**
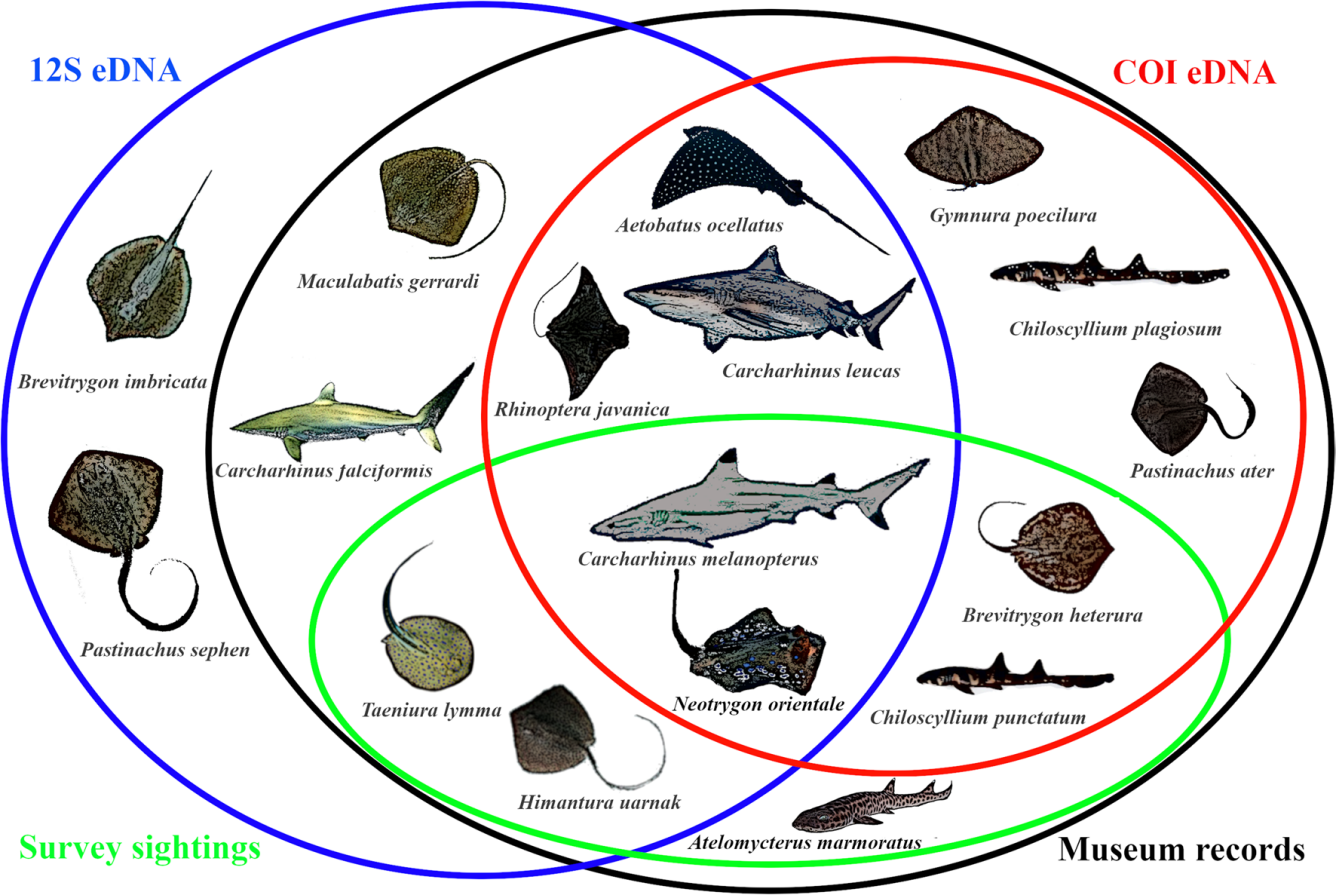
Overlapping elasmobranch diversity in Singapore compiled from historical museum records (black), contemporary sightings (green), as well as species detected by eDNA metabarcoding of 12S (blue) and COI (red). Note that *Neotrygon orientale* is part of a species complex awaiting taxonomic revision [80]

From all 84 water samples, eDNA recovered a total of 47 MOTUs consisting of 21 shark and 26 ray taxa that were identifiable to Chondrichthyes when matched against sequences on GenBank (≥ 90% for 12S and ≥ 85% for COI; Additional files 2, 3). Sixteen of the 47 MOTUs, comprising five shark and 11 ray species, had species-level matches (≥ 98.3% for 12S and ≥ 97% for COI), comprising 11, 10 and 5 MOTUs detected with 12S, COI and both markers, respectively (Fig. 2). Twenty-two MOTUs had 100% identity BLAST matches to multiple species in the GenBank database (e.g. *Carcharhinus* spp.; [53, 54]; Additional file 2) and were not analysed further.

**Fig. 3:**
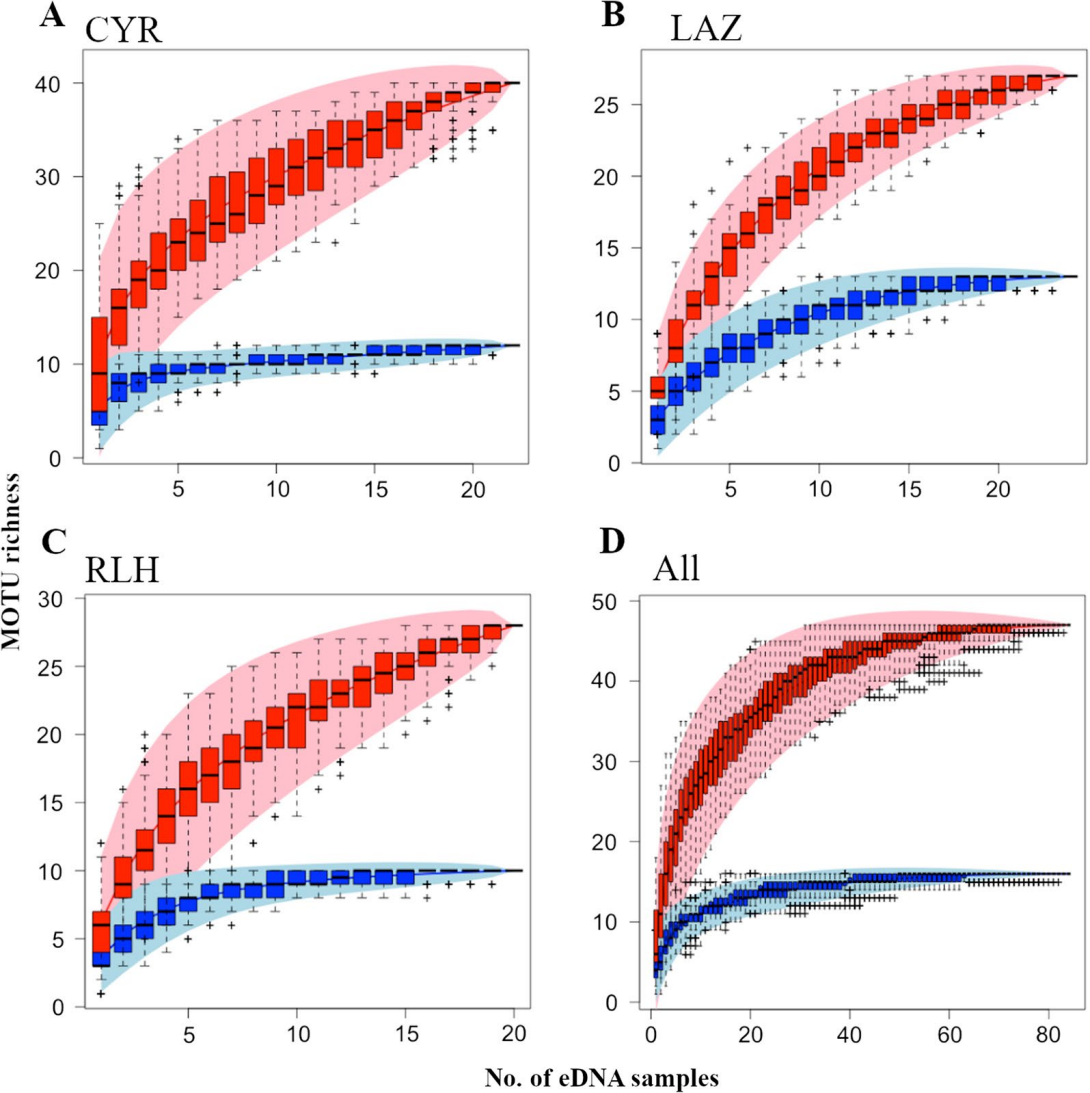
Accumulation curves of MOTUs in water samples from **A** Cyrene Reefs (CYR), **B** Lazarus Island (LAZ), **C** Raffles Lighthouse (RLH), and **D** all sites. Pink curves represent all MOTUs, while blue curves represent only MOTUs with species-level identities. Shaded areas denote confidence intervals and boxplots show standard deviation

Three sampling localities (CYR, RLH and LAZ) with the highest number of water samples (n ≥ 22) had species accumulation curves plateauing at 10–15 eDNA samples for species-identified MOTUs but continuing to increase for the all-MOTUs curve (Fig. 3A–C). While non-saturation in the total MOTUs showed that 20 water samples were insufficient to capture elasmobranch diversity at each site, species accumulation appeared to saturate when all eDNA water samples across nine localities were analysed together (*n* = 84; Fig. 3D). The species-identified MOTUs curve plateaued at 16 MOTUs with ~ 50 water samples, while the total MOTUs curve plateaued at 47 MOTUs with ~ 70 water samples.

**Fig. 4:**
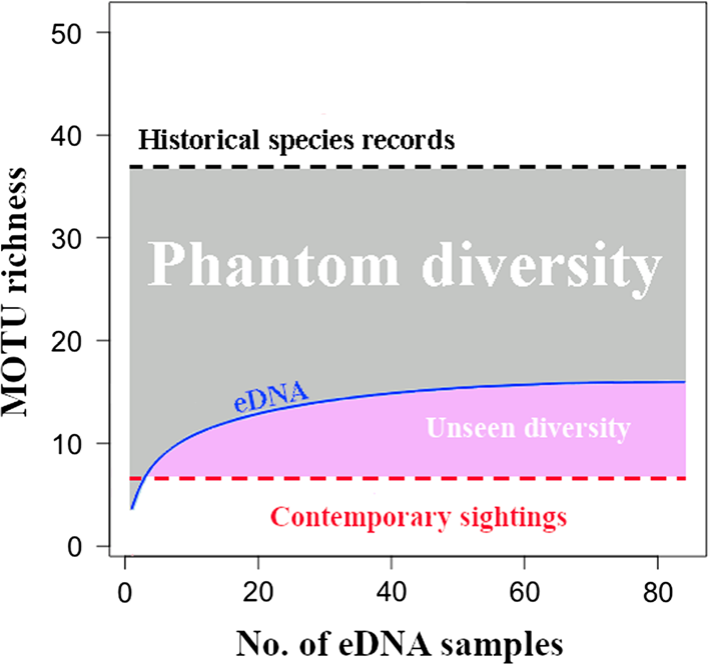
Phantom diversity decreases with eDNA sampling, which uncovers the unseen diversity not detected by conventional methods such as specimen collection and visual survey. Note that one of the 17 contemporary records (contemporary sightings or eDNA), *Atelomycterus marmoratus* (coral catshark), was not detected by eDNA

Historical records of elasmobranchs in Singapore comprised 37 elasmobranch species. Only seven species—three sharks and four rays—were sighted over the last two decades (Fig. 2, Additional file 2). The remaining 30 species from historical records not sighted recently constituted the pre-eDNA phantom diversity, amounting to 81.1% of the species records (Fig. 4). Our eDNA approach detected 16 species, including 14 overlapping with historical records (Additional file 2); and of the seven contemporary sightings, eDNA could detect all species except *Atelomycterus marmoratus* (Fig. 2; Additional file 2). Eight historically recorded species with no contemporary sightings that were rediscovered by eDNA represented ‘unseen diversity’ (Fig. 4; [28]). Therefore, the total number of contemporary records (contemporary sightings or eDNA) matching historical records was 15, up from seven pre-eDNA. Twenty-two species that were historically recorded but remained unaccounted for (even with eDNA) made up the post-eDNA phantom diversity at 59.5% of the historical species records (Fig. 4).

Two species-identified 12S MOTUs that were not recorded prior to this study are potential new records for Singapore. They are two ray species (*Brevitrygon imbricata* and *Pastinachus sephen*) that were either found to have relatively high sequence read counts (133–1038, Additional file 2) or detected frequently among sites (*Brevitrygon imbricata* with multiple detections at LAZ; *Pastinachus sephen* at CYR and LAZ; Additional file 3).

### Distribution patterns and relative abundances

**Fig. 5:**
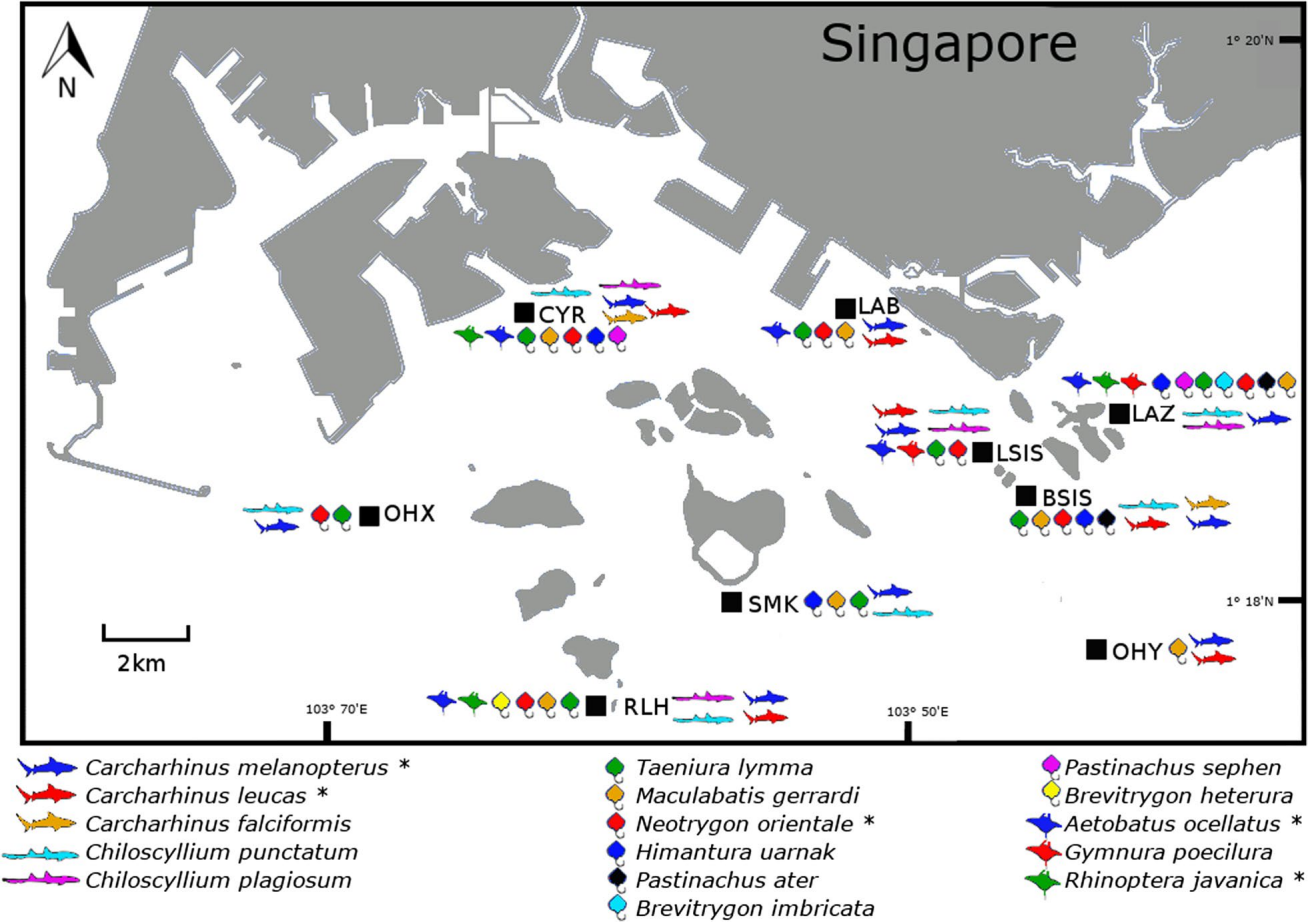
Map of sampling locations in Singapore marked with species detected via eDNA. Shark and ray icons refer to the 16 species detected at each locality. ‘*’ indicates species recovered by both 12S and COI metabarcoding. (Adapted from https://commons.wikimedia.org/wiki/File:Singapore_Outline.svg)

Among the nine localities examined, Lazarus (LAZ; *S* = 13), Cyrene Reef (CYR; *S* = 12) and Raffles Lighthouse (RLH; *S* = 10) had the highest total shark and ray diversity for the 16 MOTUs with species-level matches (Fig. 5). LAZ also recorded the highest ray diversity (*S* = 7), whereas CYR had the highest shark diversity (*S* = 5). The most commonly detected elasmobranchs were *Carcharhinus melanopterus* and *Taeniura lymma*; the former was detected across all nine sampling localities, while the latter was found in eight localities except Open Habitat Y (OHY). Among the 31 unnamed MOTUs, *Carcharhinus* sp. 9 had the highest occurrence among sites (all 9 sites; Additional file 3) while *Carcharhinus* sp. 8 had the highest sequence read counts (up to 86,915; Additional file 2). Three other ray MOTUs, *Himantura* sp. 1, *Himantura* sp. 3 and *Neotrygon* sp. 3 also recorded high sequence read counts and/or abundance across sites.

**Fig. 6:**
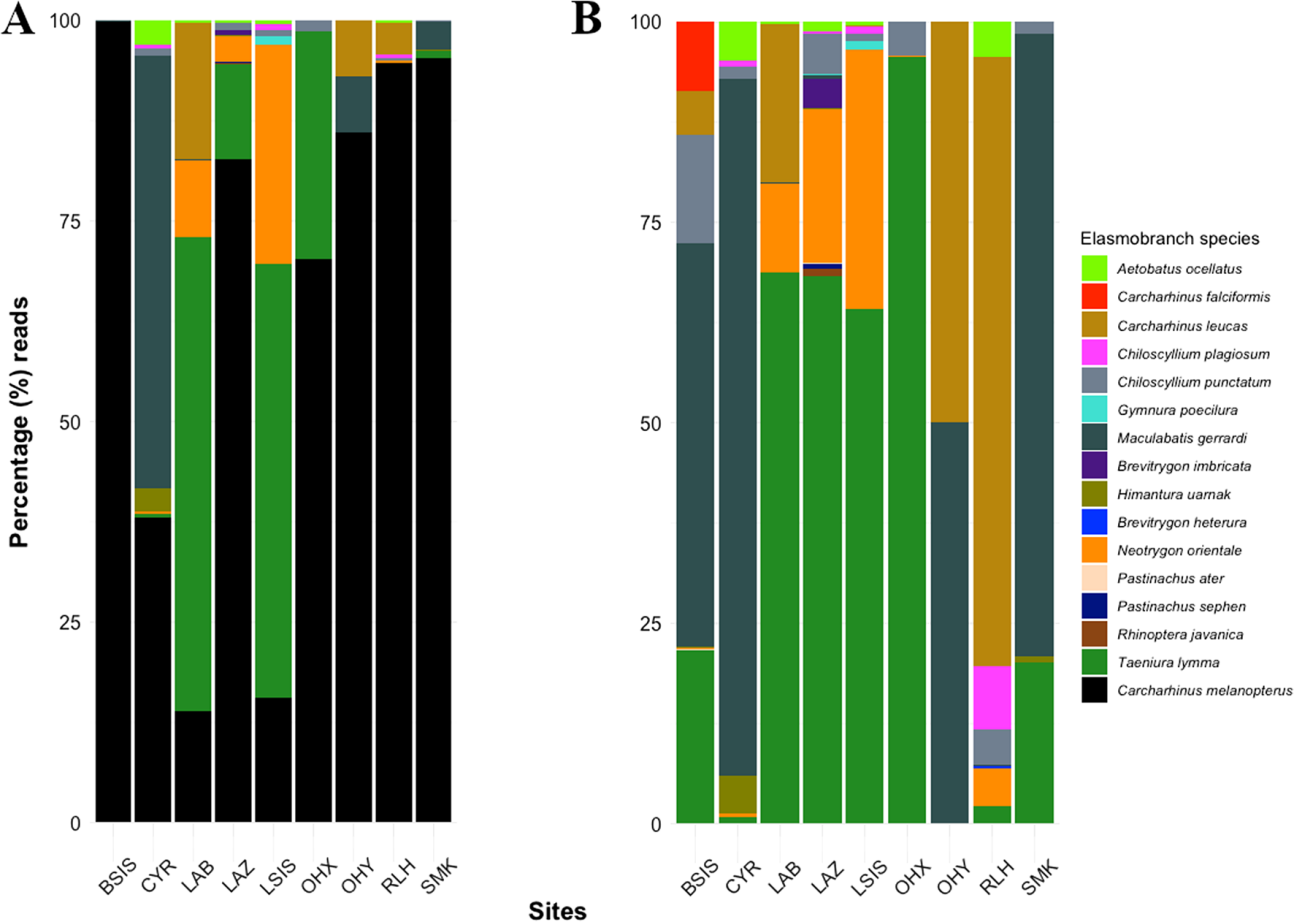
Relative abundances of eDNA reads with species-level identification for all 16 elasmobranch species (**A**), and 15 species without *Carcharhinus melanopterus* (**B**)

The relative abundances of the 16 MOTUs with species-level matches varied considerably between and within sites (Fig. 6, Additional file 4). Six of these species were found to be highly abundant overall, accounting for 99.3% of all elasmobranch reads. In particular, *Carcharhinus melanopterus* reads were the most dominant at 6 of 9 sites (≥ 70.2%), although the remaining sites also had high abundances (13.9%, 15.6% and 38.1% of reads at LAB, LSIS and CYR respectively). The latter sites were dominated by unique reads from ray species—*Maculabatis gerrardi* at CYR and *Taeniura lymma* at Little Sister’s Island (LSIS) and Labrador Beach (LAB). *Neotrygon orientale*, *Carcharhinus leucas* and *Chiloscyllium punctatum* were also fairly abundant among sites (Fig. 6). The remaining 10 species had much lower abundances.

## Discussion

### eDNA reduces phantom diversity and uncovers potential new records

A total of 37 cartilaginous fish species have been recorded previously through natural history collections and visual observations in Singapore (Additional file 2). Only seven species have been sighted over the last two decades (Fig. 2, Additional file 2), which could be due to the poor water visibility [55] that makes visual detection challenging [47]. The pre-eDNA phantom diversity was therefore relatively high at 81.1% (30 species). We applied an eDNA approach and detected 47 shark and ray MOTUs, including 16 named species, from 84 water samples collected over a 3-year period. We detected nearly half of the named species in the historical species records, uncovered more than twice the diversity of species (*S* = 15) relative to contemporary sightings of seven species, and in turn reduced the phantom diversity to 59.5% (Fig. 4, Additional file 2). Our results demonstrate that although a significant proportion of elasmobranch diversity has been missing from species records based on decades of visual sightings and conventional surveys, eDNA has revealed that these previously missed species remain present in Singapore’s waters.

Collectively, the 84 eDNA samples captured considerable elasmobranch diversity, with 47 MOTUs or putative species, suggesting that more species are present than known from Singapore (*S* = 37). The rapid saturation of the species accumulation curves for species-identified MOTUs exemplified the remarkable ability of eDNA to recover well-studied species. However, this low number of known species (*S* = 16) relative to all MOTUs (*S* = 47) demonstrated that there remain insufficient DNA barcodes covering a wide range of elasmobranch species for taxonomic matching of eDNA metabarcodes. These unidentified MOTUs could be new records or understudied taxa that are yet to be species-identified with proper reference DNA barcodes. More integrative taxonomic efforts in documenting biodiversity will help reduce phantom diversity even further, uncover new records and species, and expand databases to enhance eDNA biomonitoring [56–58]. More broadly, diversity estimates using eDNA can help drive targeted surveys in discovering and documenting more potential species in the species pool, and are also beneficial for providing updated richness estimates in areas that are expected to be more speciose but challenging to monitor.

To this end, we detected in the eDNA samples two species with no matching historical data and are potential new records for Singapore (Fig. 2)—*Brevitrygon imbricata* and *Pastinachus sephen* (Additional file 2). Earlier reports from Singapore under different names—*Trygon imbricata* and *Hypolophus sephen*—could have alluded to these species [59, 60]. Although these taxa underwent recent taxonomic revisions [61, 62], we have been unable to confirm their presence as there were no voucher specimens deposited at Singapore’s Lee Kong Chian Natural History Museum (LKCNHM). Nevertheless, our eDNA sequences matched barcodes from samples that were collected from known ranges of *B*. *imbricata* and *P. sephen* [43]. These two MOTUs also have high sequence read counts (Additional file 2) or were detected at multiple sites (Fig. 5; Additional file 3), suggesting that their detection was unlikely an artefact of sequencing error. Moreover, Singapore is within the known natural distribution ranges of these two species [61, 62], supporting them as potential new local records. We emphasise that false positive detections cannot be ruled out completely in the HTS workflow [63], and thus encourage future work to validate the unseen diversity revealed here (Figs. 3, 4, species outside green circle).

### Enhancing species detection and resolution

While there are apparent advantages of eDNA metabarcoding—including its non-invasiveness and minimal reliance on manpower—critical methodological considerations such as locus and primer choices remain [64–66]. PCR primers and the targeted genes determine the taxonomic resolution at which eDNA metabarcodes can be identified. Our previous study found that universal metazoan primers were less efficient in elasmobranch species detection, with only one shark species found (*Carcharhinus melanopterus*; [47]) despite having 26 of 84 overlapping water samples. We therefore recommended taxon-specific metabarcoding primers for higher detection success of target species of interest [47], which we demonstrate here for 16 species-identified elasmobranchs. The shark-specific COI primers used here amplify a 127-bp region of a widely-used barcoding gene that has large reference databases (e.g. GenBank, BOLD) for sequence matching [65, 67, 68] and for which there is sound understanding of its evolution [69–71]. However, these primers may be less efficient at amplifying a broad range of elasmobranch species and the targeted sequences may not have sufficient taxonomic resolution to delimit certain elasmobranch groups [28, 38, 72]. While COI primers with degenerate bases have been shown to increase the coverage of elasmobranch detection [73], the use of a single primer set can bias eDNA results and multiple markers should be used instead [74]. Therefore, the 12S rRNA region, which also has comprehensive coverage in the reference databases [75], was amplified in this study for the broadest possible taxonomic coverage (see [64, 65]).

The ambiguity of identification resulting from 100% matches of some sequences to multiple species highlights either possibilities of matching to reference sequences that were incorrectly identified, or limited resolution of 12S and COI for identifying a few elasmobranch taxa. Closer scrutiny of GenBank records is recommended to discern if these sequences match database entries that have been accurately identified and tagged with updated taxonomic names. To address the latter, alternative primers can be designed for longer target fragments (> 200-bp) to increase taxonomic resolution [76, 77]. Furthermore, conventional DNA barcoding research can prioritise these groups for database expansion, targeting alternative gene fragments which could yield higher resolution for species delimitation, including cytochrome b [78], control region [79] and NADH dehydrogenase subunit 2 [80]. These measures could aid in detecting ‘expected’ species at localities where they have been recorded and to preclude false negatives. Ultimately, improving primer design, targeting longer gene fragments (300–400-bp; [66]) and regions with higher variability will help enhance the taxonomic resolution of species detection, thereby harnessing the full potential of eDNA to better complement conventional methods in the conservation, management and biomonitoring of sharks and rays.

### Local conservation and management of elasmobranchs

Compared to earlier studies focusing on shark detection in more pristine marine environments [28, 81], our study targeted elasmobranch species in an anthropogenically-impacted environment where shark diversity was expected to be low [38]. Indeed, our eDNA results detected just five sharks but 11 ray species across multiple localities (Fig. 2). These more than double the contemporary records of elasmobranchs in Singapore, suggesting that some diversity of elasmobranch fauna remains, albeit cryptically. This may likely be the case for other highly urbanised coastal environments as well. Moreover, the phantom diversity in Singapore has been reassessed here and is now lower (59.5%) than before eDNA was applied (81.1%; Fig. 4). Of the remaining 22 undetected species, only three—*Telatrygon biasa*, *Temera hardwickii* and *Urogymnus granulatus*—do not have either 12S or COI sequences on GenBank. Implicitly, these species could have remained undetected by eDNA due to absence of reference sequences for species matching. Further investigations are needed for the 19 unaccounted species to discern if absences are due to limits of present eDNA methods or from local extirpations.

Formulation of conservation and management strategies typically require sightings data for taxa of concern [12, 82]. This is challenging for elasmobranchs in urbanised, turbid environments such as Singapore due to the low water visibility. eDNA presents an alternative and viable method for monitoring multiple shark and ray species simultaneously, especially in areas where visually-reliant methods such as BRUVs, UVCs and drones are less effective. The ability of eDNA to complement visual methods will enable researchers to better assess the extent of declines and absences. To ascertain that the absences are due to actual extirpations and rule out learned avoidance behaviour of sharks in urban environments [16], we suggest integrating eDNA with visual methods to validate these possible losses so as to chart suitable policy pathways for management and restoration of elasmobranch populations [12].

Apart from being an alternative sampling method with more consistent detection results, the utility of eDNA goes beyond simply reducing phantom diversity [14, 28]. Accurate assessments of relative abundances are often thought to be challenging with most biases stemming from the use of universal primers for broad metabarcoding work [83], but it has become increasingly evident that using a multi-marker approach with several species-specific primers could effectively circumvent this issue [51, 74]. In particular, we used a combination of shark- and vertebrate-specific primers to infer relative abundances [84] of elasmobranchs in Singapore, data that are essential for robust management efforts. Mapping species distributions and estimating relative abundances of elasmobranch eDNA signals (Figs. 5, 6) highlighted potential diversity hotspots which are of conservation concern. For example, our data show that sites like CYR, LAZ and RLH host richer assemblages of sharks and rays, corroborating existing knowledge from citizen science records [49] that could lend further support for site protection measures. Furthermore, eDNA analysis here has expanded the local ranges of two ray species (*Neotrygon orientale* and *Brevitrygon heterura*), previously reported only along Singapore’s northern coastline [49], to now include areas south of the main island (Fig. 5).

Despite overwhelming evidence supporting the viability of normalised read counts for abundance estimates [85], we acknowledge the associated limitations where eDNA can only provide rough assessments of relative abundances [83]. While biases from complex eDNA dynamics such as shedding rates between species remain to be addressed, body mass has been demonstrated to positively correlate with read counts especially for larger-sized organisms with higher DNA shedding rates [86], such as the elasmobranchs in this study [8]. The correspondence between relative abundance and frequency of visual observations (Additional file 4) also supports eDNA for quantitative measures [87]. Nevertheless, approaches such as the Hellinger transformation of read counts can enhance eDNA’s reliability for quantifying abundances, and it must be emphasised that normalisation is required at the very least to avoid biased inferences [88]. Another potential workaround for improving abundance estimates involves the addition of internal DNA standards, where known DNA concentrations of non-target species are included into eDNA samples for copy number correction [89]. Collectively, these strategies can increase the confidence of quantifying abundances from eDNA metabarcoding results.

Relative abundance estimates of species that are of conservation concern can facilitate projections of their habitat preferences [90]. For instance, we detected six ‘Vulnerable’ species according to IUCN Red List of Threatened Species (IUCN 2020)—*Aetobatus ocellatus*, *Carcharhinus falciformis*, *Carcharhinus melanopterus, Himantura uarnak*, *Maculabatis gerrardi* and *Rhinoptera javanica*. We found three possible residency hotspots, such as BSIS for *C. falciformis*, LAZ for *R. javanica*, and CYR for *A. ocellatus, H. uarnak* and *M. gerrardi*, supporting more stringent site protection to conserve these threatened elasmobranchs. The current inability of eDNA methods to discern body sizes, sex and developmental stages of organisms [28] remains a key limitation in this case. Nevertheless, eDNA methods are ideal components of a comprehensive monitoring toolkit that can provide spatial information critical for formulating actionable management plans and policies [84, 91, 92].

## Conclusion

We have here demonstrated the utility of eDNA detection of sharks and rays from HTS of seawater samples. By comparing our results with natural history collections and visual survey reports, we show that eDNA metabarcoding of seawater samples for elasmobranch detection in Singapore is a more viable and consistent approach to monitor these elusive species over survey sightings. Despite substantially reducing the phantom diversity of sharks and rays, the number of undetected yet expected species remains high (*S* = 22). On the one hand, these may represent dark diversity, or true local extinctions due to species’ inability to thrive in a hyper-urbanised coastal environment. On the other hand, it could mean that a large number of elasmobranchs remain as phantom species in Singapore, having successfully evaded detection thus far, highlighting the urgent need to improve our biomonitoring methods so as to better understand and manage the numerous threats against elasmobranch populations here [28].

eDNA metabarcoding methods have shown enormous promise for complementing conventional methods in biomonitoring, species discovery and conservation applications. Emerging platforms can further propel eDNA’s utility and relevance in these fields by providing opportunities for real-time eDNA metabarcoding with nanopore sequencing [81], improved species specificity with hybrid capture metabarcoding [93], as well as field detection with loop-mediated isothermal amplification assays (LAMP; [73]) and the ‘Specific High-sensitivity Enzymatic Reporter un-LOCKing’ method (SHERLOCK; [94, 95]). These novel approaches can be easily incorporated into field-ready laboratories for mobile biomonitoring [57]. Besides improving the design of higher-resolution primers to eradicate false negatives from the current eDNA experimental design, application of these new techniques may address many of the limitations here. For instance, the heightened single-species specificity with LAMP [73] can be used to search for the missing coral catshark (Fig. 2, Additional file 2) and for validating the presence of MOTUs detected solely by eDNA. The continual development and application of sensitive detection methods will further reduce phantom diversity and enhance our confidence in species absences and local extinctions.

Beyond the abovementioned uses of eDNA, we are learning more about its applicability to more extensive and varied research problems, such as interpreting sequences for metaphylogeography [96] and studying intraspecific diversity [97]. With rapid advancements in detection technologies and increasingly diversified applications, eDNA is likely to play an increasingly significant role in biomonitoring, management and conservation, especially of threatened taxa and habitats.

## Methods

### Water sampling and processing

A total of 84 2-L water samples were collected from the subtidal and intertidal areas at nine localities south of mainland Singapore between March 2017 and April 2019 (Fig. 5), of which 26 samples were from a previous study [47]. Subtidal sites were sampled at two depths, 1 m (shallow) and 10 m (deep) from the sea surface. The localities represented a variety of coastal environments, including coral reefs, seagrass, mangroves and open water habitats.

All water samples were collected under clear weather conditions. For subtidal sites, 2-L water samples were collected from a 5-L Van Dorn water sampler. Intertidal samples were collected by hand using two sterile 1-L bottles at each of two sampling points that were at least 10 m apart, starting with the first collection at a downstream position relative to the current and moving upstream for the second collection to reduce chances of contamination from the collectors. Water samples were kept on ice, transported back to the laboratory for vacuum-filtering through sterile nylon filter membranes (Thermo Scientific; diameter, 47 mm; pore size, 0.22 μm) in autoclaved filter units, and subsequently stored at − 80 °C. The time from collection to storage took < 2 h.

Contamination control measures included cleaning of all working surfaces, laboratory apparatus and sampling equipment with 20% household bleach diluted with Milli-Q water. All collection bottles and filtration equipment (filter units and membranes) were autoclaved and disposable gloves were also disinfected with 20% bleach prior to use. All post-filtration work was performed in a biological safety cabinet. Negative controls for field collection, DNA extraction and polymerase chain reaction (PCR) were set up and processed in the same way as the samples to identify potential contamination; we used molecular-grade water in place of template DNA for the negative controls.

### eDNA extraction, amplification and sequencing

Filter membranes were first incubated in 900 μL CTAB (hexadecyltrimethylammonium bromide) with 20 μL of 20 mg/mL proteinase K for 3 h at 55 °C. The digest was then purified via phase separation with phenol:chloroform:isoamyl-alcohol (25:24:1) and incubated in 60% isopropanol for 16 h at − 30 °C to increase DNA recovery and yield.

For the metabarcoding assay, we amplified two gene fragments—12S-V5 ribosomal RNA and cytochrome *c* oxidase subunit I (COI). The 12S-V5 locus (85–117-bp amplicon) was amplified using the *ecoPrimers* primer set, F: 5ʹ-ACTGGGATTAGATACCCC-3ʹ, and R: 5ʹ-TAGAACAGGCTCCTCTAG-3ʹ [50]. For COI, we used two universal fish barcoding forward primers, FishF1: 5ʹ-TCAACCAACCACAAAGACATTGGCAC-3ʹ and FishF2: 5ʹ-TCGACTAATCATAAAGATATCGGCAC-3ʹ [98], along with an elasmobranch-specific reverse primer SharkCOI-MINIR: 5ʹ-AAGATTACAAAAGCGTGGGC-3ʹ [99] to amplify a 127-bp fragment (see [28, 38]). Both 12S and COI primers were respectively tagged with unique 9-bp or 8-bp sequence tags at the 5ʹ end to allow sequence-to-sample association in the downstream demultiplexing step [100]. We ensured that each reaction had its own unique sequence tag combination (for up to 96 unique tag combinations for each gene).

Five PCR replicates were performed for each gene per water sample, for a total of 840 reactions (84 samples × 5 replicates × 2 genes). Each PCR reaction mix, comprising a total volume of 25 μL, contained 0.5 μM of each primer (Integrated DNA Technologies), 0.5 μg bovine serum albumin (New England Biolabs), 25 mM magnesium chloride (New England Biolabs), 5 μL template DNA, 9.25 μL sterile water with 1U BioReady rTaq DNA polymerase with 1× reaction buffer (v/v) (Bulldog Bio Inc., China) for the 12S vertebrate primers, and 12.5 μL of GoTaq DNA polymerase for the elasmobranch COI primers. The thermal cycling profile for 12S-V5 was 95 °C for 7 min, 36 cycles of 94 °C for 30 s, 52 °C for 30 s, 72 °C for 40 s, and a final extension for 5 min at 72 °C; while for COI it was 95 °C for 15 min, 36 cycles of 94 °C for 1 min, 52 °C for 1 min, 72 °C for 1 min, and a final extension for 5 min at 72 °C. Amplification success was verified on 2% agarose gels stained with GelRed (Cambridge Bioscience).

A total of 840 tagged amplicon samples and 940 negative controls were then combined into 20 pools (up to 96 unique PCR reactions each; see above) and cleaned using 1.5–1.8× AMPure XP magnetic beads (Beckman Coulter). PCR-free library preparation was performed where each of these 20 library pools were further multiplexed with unique Illumina adapters (Set B), using NEBNext Ultra II DNA Library Prep Kit (New England Biolabs) following manufacturer’s protocol up to the adapter ligation step (i.e. no PCR enrichment). The libraries were pooled in equimolar ratios and outsourced to the Genome Institute of Singapore for sequencing over three lanes of Illumina HiSeq 4000 (151 × 151-bp paired ends), each spiked with 20% PhiX to improve base diversity. We allocated a sequencing depth of up to 1 million reads per amplicon sample to increase the detection chances of rare taxa [101].

### Bioinformatics and data analyses

Illumina paired-end reads were merged using PEAR v0.9.11 [102]. Maximum assembly length (*m*) was set at 200-bp and quality score threshold (*q*) was set at 20. Minimum assembly length (*n*) was set to 100-bp and 80-bp for 12S and COI respectively. OBITools v1.2.11 [103] was used for demultiplexing and further processing of assembled reads. As we previously observed that a poorer quality of reverse reads would affect the integrity of the reverse barcode tags and in turn, lower demultiplexing efficacy [47], we demultiplexed sequence reads to respective PCR replicates using only the unique forward primer tag. For demultiplexing via *ngsfilter*, we used the default settings, where no mismatch was allowed for barcode tags, while up to two mismatches were allowed for the primer sequences. Following which, cutadapt v1.18 [104] was used to remove the reverse primer and tag sequences. All successfully demultiplexed and primer-free reads were concatenated into a single file, sequence records grouped, and dereplicated using *obiuniq*. Finally, sequences were binned into PCR replicate files using *obisubset*.

The dataset was filtered for metazoan sequences using BLASTn implemented on BLAST+ v2.8.1 [105] to match against the NCBI *nt* database (downloaded 2nd September 2019), retaining only sequence reads with ≥ 80% sequence similarity. The output was parsed with readsidentifier v1.0 [106] to obtain preliminary taxonomic identities of each sequence. Only sequences assigned to ‘Chondrichthyes’ (≥ 80% sequence similarity) were retained for further analyses. This step also allowed us to eliminate non-target reads from the dataset.

Quality filters were applied to eliminate reads with amplification and sequencing errors, while ensuring that read coverage was comparable across samples. We implemented a read count filter for each PCR replicate based on a relative threshold. Only sequences whose abundance exceeded 0.0001 (1e^−4^) of the total read count for the PCR replicate were used in the analyses; i.e., sequences in replicates with higher read counts have to meet a higher threshold in order to be retained for analysis. To implement this filter, we used the *obistat* module to summarise the total read count per replicate file. However, in some of the 1e^−4^ datasets, we found that singleton reads had met the threshold. These were subsequently excluded by setting a minimum read count of ≥ 2 with *obigrep*, which was also set to retain sequences of lengths 90–140-bp and 80–120-bp for 12S and COI respectively. We then used *obiclean* to collapse sequences with potential PCR sequencing errors into respective unique sequence reads. Sequences from sample PCR replicates were also matched against sequences found in the negative PCRs; although none of these sequences were elasmobranch sequences, they were still deemed potential contaminants and removed from downstream analyses.

Taxonomic assignment was performed by applying respective species delimitation thresholds (class-level identity ≥ 90% for 12S and ≥ 85% for COI; species-level identity ≥ 98.3% for 12S [43] and ≥ 97% for COI [107] for each gene (Additional file 2) to collapse the unique sequence reads into MOTUs [108]. We eliminated potential false positives by removing MOTUs present in only one PCR replicate and/or taxa that have documented ranges outside the Indo-Pacific. Additionally, MOTUs that were (i) not assigned species-level identities, or (ii) matched at high percentages (99–100%) to multiple taxa within the same or different genera respectively were also removed.

To estimate elasmobranch species richness, we plotted species accumulation curves for all MOTUs and MOTUs with species-level identities against number of water samples collected using the *specaccum* function [109] of *vegan* v2.5 package [110] in RStudio (R Core Team, 2017). Sequence read counts were normalised by sample read depth to estimate the relative abundances of elasmobranch species [88].

### Compiling historical species records

Records of elasmobranch species reported from Singapore (not via eDNA) were consolidated through three different sources. First, historical records of shark and ray species (from the 1960s) were obtained from the collection at LKCNHM. Second, contemporary sightings were compiled from two decades of citizen-science reports (2000–2019), supported by in-situ images, and obtained from WildSingapore (http://www.wildsingapore.com/wildfacts; [49]). Third, elasmobranch species documented in the grey literature from 2003 to 2019 were also compiled, and these were noted to fully overlap with both historical records and contemporary sightings. We then integrated all three datasets, removing overlapping records to estimate phantom diversity before and following the application of eDNA in this study (Additional file 2).

## Supplementary Information

**Additional file 1. **Heatmap illustrating log_10_ sequence read counts for all 16 Chondrichthyes species detected by both 12S and COI across all PCR replicates.

**Additional file 2. **Known Chondrichthyes species diversity in Singapore, compiled from museum records and sighting reports marked with ‘✔’, and from eDNA metabarcoding marked with ‘➕’. Taxonomic assignments, read counts and database match information are indicated only for MOTUs detected by eDNA in this study.

**Additional file 3. **Heatmap of all MOTUs and respective percentage matches to sequences from GenBank database. See Additional file 2 for taxonomic assignments for each MOTU.

**Additional file 4. **Species abundance patterns of the six contemporary sighted species are found to be in agreement with relative abundance from eDNA, as shown in the plots of survey sightings frequency against their respective eDNA sample detection frequencies (A), and number of sites where they were seen during surveys against number of sites with eDNA detection (B).

**Additional file 5. **Sample demultiplexing information.

## Data Availability

Raw sequence reads generated in this study have been uploaded to GenBank NCBI Sequence Read Archive under BioProject PRJNA673533 (SRR15093454-SRR15093473) [111]. Additional file 5 contains the sample demultiplexing information.

## Abbreviations

BLAST: Basic local alignment search tool
BRUV: Baited remote underwater video
BSIS: Big Sisters’ Island
COI: Cytochrome *c* oxidase subunit I
CYR: Cyrene reefs
eDNA: Environmental DNA
HTS: High-throughput sequencing
IUCN: International Union for Conservation of Nature
LAB: Labrador
LAMP: Loop-mediated isothermal amplification
LAZ: Lazarus Island
LKCNHM: Lee Kong Chian Natural History Museum
LSIS: Little Sisters’ Island
MOTU: Molecular operational taxonomic unit
OHX: Open habitat X
OHY: Open habitat Y
PCR: Polymerase chain reaction
RLH: Raffles Lighthouse
SHERLOCK: Specific High-sensitivity Enzymatic Reporter un-LOCKing
SMK: Semakau
UVC: Underwater visual census
