# Supplementary figures and images for: Seeing through sedimented waters: environmental DNA reduces the phantom diversity of sharks and rays in turbid marine habitats
Source: BMC Ecol Evol. 2021 Sep 6;21:166. doi: 10.1186/s12862-021-01895-6 (PMC8422768; doi:10.1186/s12862-021-01895-6)

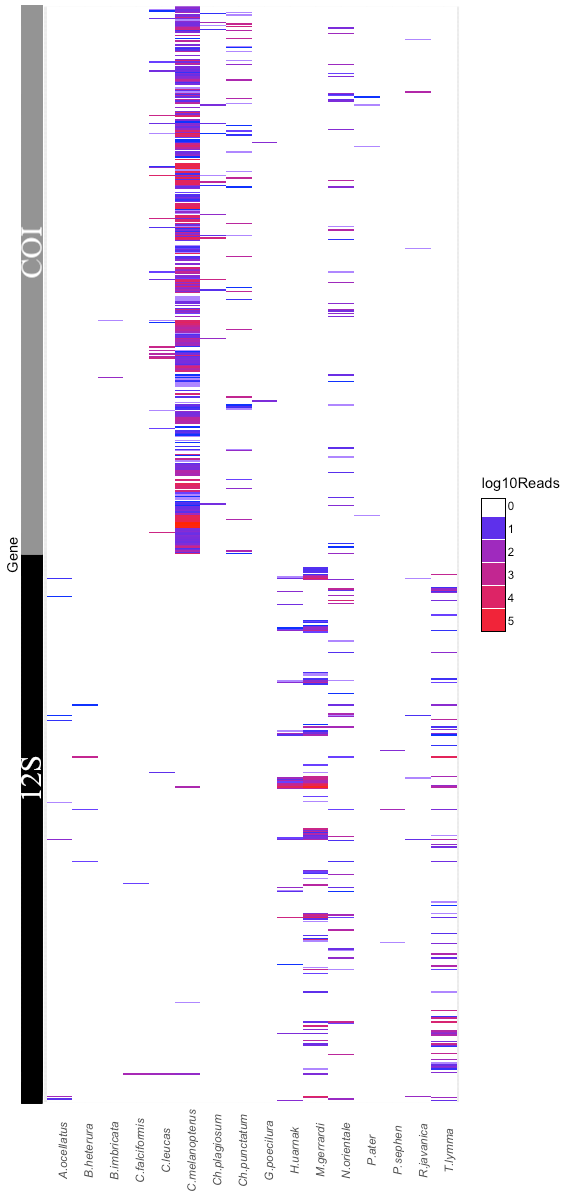

Supplement: Supplementary file 1 — Additional file 1. Heatmap illustrating log10 sequence read counts for all 16 Chondrichthyes species detected by both 12S and COI across all PCR replicates. [file 12862_2021_1895_MOESM1_ESM.tiff]

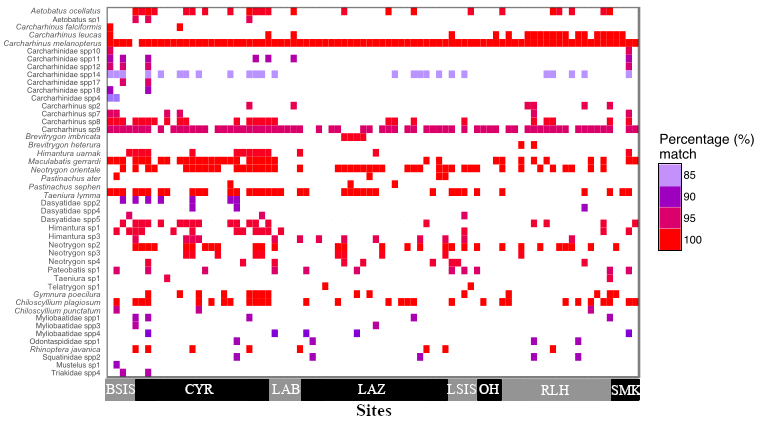

Supplement: Supplementary file 3 — Additional file 3. Heatmap of all MOTUs and respective percentage matches to sequences from GenBank database. See Additional file 2 for taxonomic assignments for each MOTU. [file 12862_2021_1895_MOESM3_ESM.tiff]

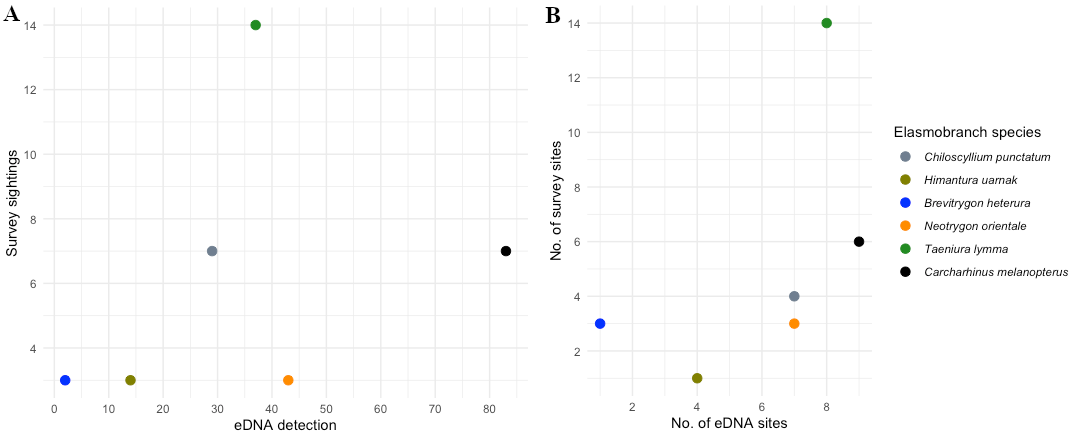

Supplement: Supplementary file 4 — Additional file 4. Species abundance patterns of the six contemporary sighted species are found to be in agreement with relative abundance from eDNA, as shown in the plots of survey sightings frequency against their respective eDNA sample detection frequencies (A), and number of sites where they were seen during surveys against number of sites with eDNA detection (B). [file 12862_2021_1895_MOESM4_ESM.tiff]
